# Percutaneous Superimposed O-Arm-MRI-Navigated Biopsy for Spinal Column Pathologies

**DOI:** 10.3390/diagnostics13132252

**Published:** 2023-07-03

**Authors:** Mohammad W. Al-Smadi, István Kozma, Siran Aslan, Balázs Bölöni, Árpád Viola

**Affiliations:** 1 Department of Neurosurgery and Neurotraumatology, Dr. Manninger Jenő National Traumatology Institution, 1081 Budapest, Hungary; smadi996@hotmail.co.uk (M.W.A.-S.); istvan.kozma88@gmail.com (I.K.); drsiran.5@gmail.com (S.A.); bolonibalazs93@gmail.com (B.B.); 2 Department of Operative Techniques and Surgical Research, Faculty of Medicine, University of Debrecen, 4032 Debrecen, Hungary; 3 Department of Neurosurgery, Andras Josa Teaching Hospital, 4400 Nyiregyhaza, Hungary; 4 Department of Neurotraumatology, Semmelweis University, 1081 Budapest, Hungary

**Keywords:** percutaneous spinal biopsy, imaging-guided biopsy, O-arm CT navigation, spinal tumors, diagnostic accuracy, biopsy efficiency, O-arm CT-MRI fusion

## Abstract

Classifying spinal tumors can be challenging due to nonspecific clinical and radiological qualities, and a precise biopsy is crucial for an accurate diagnosis and treatment planning. This study aimed to enhance the accuracy and efficiency of spinal biopsies integrating Cone Beam Computed Tomography (CBCT) and magnetic resonance imaging (MRI) modalities using an O-arm CT navigation system. Eighteen patients with different spinal lesions underwent 18 biopsies following the Stealth Station navigation system Spine 8 protocol. Preoperative MRI scans were merged with intraoperative CT navigation systems for continuous monitoring during the biopsy process. The combined imaging technique accurately identified the diseased lesion type in all biopsies, demonstrating 100% sensitivity and specificity. In conclusion, combining MRI and CT imaging modalities significantly improved spinal biopsy accuracy and efficiency, differentiating between pathological entities. However, large-scale studies are desired to validate these findings and investigate potential benefits in different clinical scenarios. Although this method requires general anesthesia, its potential profits in avoiding misdiagnosed lesions and decreasing the need for further invasive procedures make it a promising approach for improving spinal biopsy accuracy and efficiency.

## 1. Introduction

Advanced imaging techniques such as computed tomography (CT) and magnetic resonance imaging (MRI) have greatly facilitated the identification of spinal abnormalities [1]. However, specific spinal lesions may exhibit nonspecific clinical and radiological features and a confirmatory diagnosis for appropriate treatment is needed [2]. A precise biopsy is crucial in accurately diagnosing neoplastic lesions in such cases.

Open spine procedures have traditionally been considered the gold standard for biopsy; despite their invasiveness and higher morbidity rates [3], it typically provides sufficient tissue for histological and immunohistochemical analysis, resulting in a highly informative procedure [4,5,6]. Nevertheless, the challenges associated with open biopsy complications have spurred the need for improved biopsy techniques. Closed biopsy techniques, including fine-needle aspiration and image-guided percutaneous spine biopsy, have emerged as promising alternatives, offering advantages such as reduced invasiveness and lower cost [7,8]. Subsequent advancements in closed biopsy included the utilization of local anesthesia and incorporating nerve root monitoring, further enhancing the procedure [9]. However, ensuring improved accuracy remains a crucial challenge to establishing closed biopsy as a superior technique to open biopsy [10]. 

Fluoroscopy, ultrasound, CT, and MRI have been commonly used imaging modalities for guiding percutaneous spine biopsies [6,8,11,12,13]. While fluoroscopy has its advantages, providing real-time imaging, it may have limitations in accurately visualizing vital structures surrounding the lesion and paravertebral lesions [10,14]. On the other hand, CT offers more precise imaging of these structures, particularly in challenging areas such as the upper thoracic and cervical spine [10]. However, there have been cases where biopsy procedures had to be repeated due to inadequate sample tissue or necrotic parts of the lesion [13]. Consequently, researchers needed to prioritize the development of a more accurate diagnostic method.

This study aims to enhance the accuracy, efficiency, and adequacy of spinal biopsy procedures by integrating CT and MRI modalities using an O-arm CT navigation system. Our objective is to develop a comprehensive and reliable approach that addresses the limitations of individual imaging techniques, enabling the procurement of viable, nonnecrotic tumor samples for accurate diagnosis and subsequent treatment planning.

## 2. Materials and Methods

The study population consisted of 18 patients with different spinal tumors who were scheduled to undergo surgery. The Stealth Station navigation system Spine 8 (SSS8) (Medtronic, Medtronic Sofamor Danek, Minneapolis, MN, USA) protocol was followed, and a gadolinium contrast MRI scan of the spinal segment was performed using the SIGNA™ Voyager 1.5T MRI machine. 

After informed obtained consent, all biopsies were performed under general anesthesia, and the patients were positioned in the prone position on a Maquet carbon operating table. The O-arm device was sterilely isolated using the Sterile Tube Drape O-arm System.

The Spine Stealth Air reference frame (RF) was attached to the spinous process above the target vertebral body. The O-arm registration menu was prepared in the Cranial software on the SSS8. The navigation camera was used to ensure that the O-arm trackers and the RF were visible, and the O-arm device was connected to the SSS8 via an Ethernet cable. 

### 2.1. Imaging Procedure

The first step of the imaging procedure involved taking O-arm scans of axial and sagittal planes to verify the target area, followed by a three-dimensional (3D) scan of the selected spinal region. For the lumbar spine, 3–4 levels were scanned; for the thoracic spine, 4–5 levels were scanned. After verification, the 3D scan was performed with the O-arm device. Later, 3D scans were registered and automatically transmitted to the SSS8. Then, in the images menu of the SSS8 Cranial software, the patient’s O-arm and MR scans performed according to the SSS8 protocol were selected.

### 2.2. Images Processing and Analysis

In the “Merge Images” menu, the O-arm and MR scans were merged manually using the “Manual Merge” function. Then the “Verify Merge” option was selected to confirm the merging process of the O-arm and MRI scans. The memory function of the O-arm was used to determine the scan and parking positions of the O-arm device. The navigating instruments were registered on Patient Reference, including the Passive Planar Blunt Probe, SureTrak II Large Clamp, and SureTrak II Small Passive Tracker Orange. The SureTrak II Large Clamp was attached to the Johnson and Johnson 8G Vertebroplasty Needle. The SureTrak II Small Passive Tracker Orange was linked to the SureTrack II Large Clamp.

### 2.3. Biopsy Procedure

**Figure 1:**
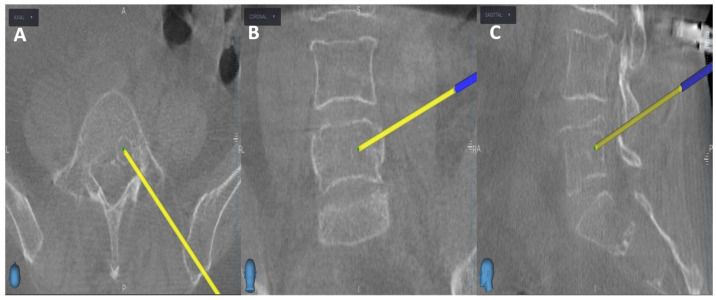
Intraoperative O-arm scan; the yellow projection line indicates the entry point for the needle biopsy: (**A**) Axial view; (**B**) Coronal view; (**C**) Sagittal view.

**Figure 2:**
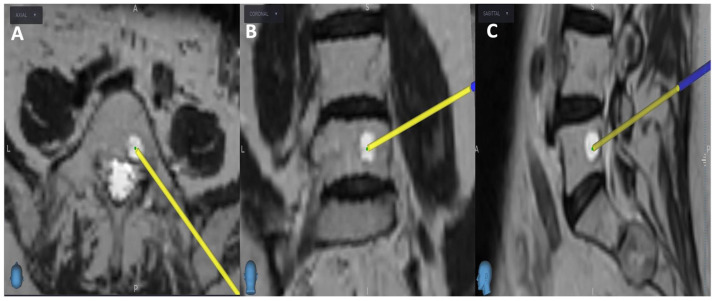
MRI scan; the yellow projection line displays the targeted biopsy site for the needle biopsy procedure: (**A**) Axial view; (**B**) Coronal view; (**C**) Sagittal view.

Initially, the entry point is determined through the O-arm scan (Figure 1). Next, the target point for sampling is set using an MRI scan (Figure 2). Afterward, the surgical plan for the needle biopsy is progressed by utilizing the instrument projection function.

**Figure 3:**
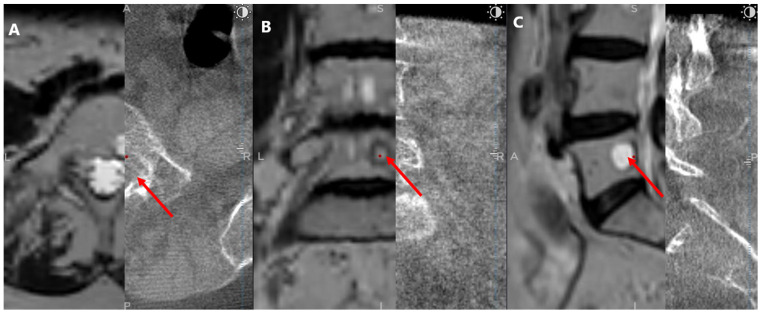
Postbiopsy O-arm and MRI fused image: (**A**) Axial view; (**B**) Coronal view; (**C**) Sagittal view. The left half of each view displays the MRI scan. The right half displays the O-arm scan. Red arrows indicate the biopsy sample location.

After a 5 mm skin incision, the surgical technique projected on the screen entered the vertebral body and performed a needle biopsy sampling using the Johnson and Johnson 8G Vertebroplasty Needle. During vertebroplasty sampling, the position of the needle to be inserted was also checked on the O-arm scan, not only on the MRI scan. After the needle biopsy sampling, the O-arm device was returned to the previous O-arm scanning position. Next, an O-arm scan of the same spinal segment was performed, and then the O-arm scan was merged with the needle biopsy plan previously selected in the MRI scan using the “Merge Image” function. If the position of the needle biopsy channel in the second O-arm scan matched the needle biopsy plan selected in the MRI scan, the needle biopsy sampling was considered successful (Figure 3). Finally, the Spine Stealth Air reference frame was removed.

### 2.4. Measurement and Statistical Analysis

**Figure 4:**
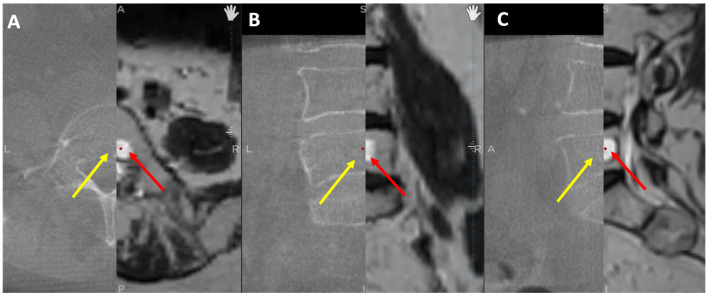
O-arm and MRI fused images: (**A**) Axial view; (**B**) Coronal view; (**C**) Sagittal view. The left half of each view displays the O-arm scan. The right half displays the MRI scan. Red arrows indicate the tumors and yellow arrows show where the tumor should be visible.

To validate that CT images were improved through merging with MRI images, the tumor dimensions were measured separately on axial, sagittal, and coronal planes in cases where the tumor was found in both modalities. While MRI scans revealed tumors in all 17 patients, CT scans revealed tumors in only 8 cases, implying that some tumors were not visible on CT (Figure 4). As a result, we could only compare tumor dimensions in these 8 cases. GraphPad Prism 9 software was used to perform statistical analysis with a significance level set at *p* ≤ 0.05. Normality was checked for all data distributions, and a Student’s *t*-test was employed depending on the results. The Pearson correlation coefficient was also calculated to examine the correlation between data sets.

## 3. Results

In this study, we conducted 18 biopsy procedures on 18 patients to obtain viable portions of suspected lesions for cytological examination (Table 1). The procedures included ten lumbar, one sacral, and eight dorsal biopsies. For 16 out of the 18 patients, PET-CT FDG imaging was initially performed to identify the lesions; however, for all patients, we followed up with MRI investigations due to low resolution and sensitivity. The remaining two patients underwent MRI imaging directly. Contrasting with the remaining ten biopsies that had no previous attempts, seven (four lumbar and three dorsal) had prior negative cytology results from C-arm freehand guided biopsies.

Among the 18 patients, 13 had confirmed primary malignancies. Of these, seven cases were breast cancer, with three patients having an additional primary tumor (two adenocarcinomas and one renal cell carcinoma). A total of two cases involved ovarian squamous cell carcinoma and prostate cancer. The remaining four cases were non-Hodgkin lymphoma, including two follicular lymphomas and two diffuse large B-cell lymphomas. 

All 18 biopsies yielded positive results, confirming metastasis in 13 patients with previously identified tumors. In addition, among the three patients with an additional primary tumor alongside breast cancer (two adenocarcinomas and one renal cell carcinoma), two were found to have breast cancer metastasis, while one was diagnosed with lung adenocarcinoma. Among the remaining five patients without known primary tumors, one had follicular non-Hodgkin lymphoma, two had monoclonal gammopathy of undetermined significance, one had a hemangioma, and one had a benign synovial cyst.

**Figure 5:**
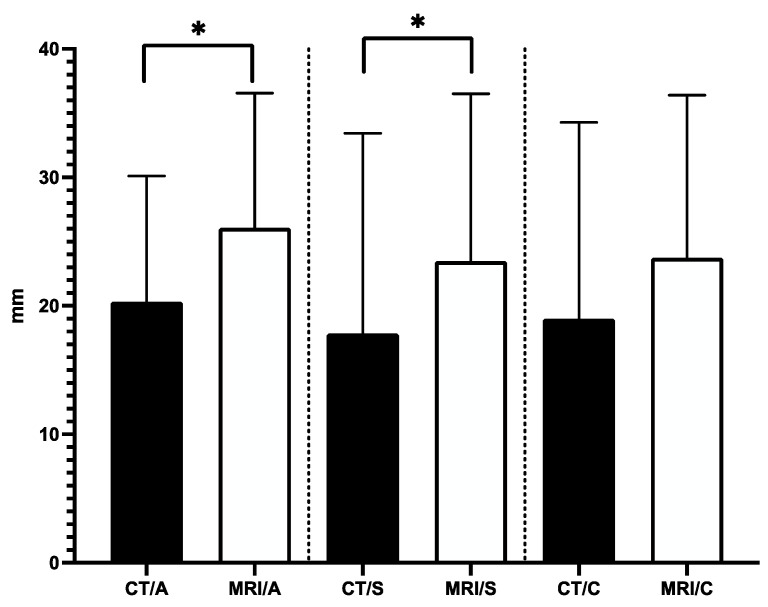
Comparison of Tumor Dimensions in CT and MRI. *n* = 9, mean ± SD, * *p* < 0.05 vs. CT.

Our analysis showed that the average tumor dimensions in the CT axial, sagittal, and coronal planes were 20.3 mm (±9.7), 17.8 mm (±15.6), and 18.9 mm (±15.2), respectively. On the other hand, the mean tumor dimensions in the MRI axial, sagittal, and coronal planes were 26 mm (±10.4), 23.4 mm (±13), and 23.7 mm (±12.6), respectively. The variations in the measured tumor dimensions between CT and MRI in the axial and sagittal planes were statistically significant (Figure 5).

## 4. Discussion

The examination of spinal lesions, whether infectious, tumor-related, or of other origins, has been dramatically enhanced by CT and MRI imaging. This advancement primarily provides a detailed visualization of the anatomical extent and identification of the active regions of the lesion following medium contrast injection. However, the lack of specificity in these imaging techniques frequently prevents a conclusive diagnosis of the underlying cause when based only on radiographic findings. Consequently, histological analysis is typically necessary to accurately diagnose most spinal tumors and pseudotumorous lesions.

Open biopsy has long been regarded as the gold standard for obtaining tissue samples for histological analysis due to its perceived accuracy and adequacy. However, the significant complications associated with open biopsy have led to exploring alternative approaches, notably closed biopsy techniques. In recent years, notable advancements have been made in closed biopsy, with a critical focus on incorporating imaging modalities to ensure precise and sufficient sampling from the target tissue. The outcomes of many studies have been remarkable, demonstrating the potential of closed biopsy to overcome the challenges associated with open biopsy while retaining its advantages. Nevertheless, these convincing results justify the need for further exploration for a technique that balances accuracy, adequacy, and minimizing complications. Fluoroscopy, ultrasound, CT, and MRI have been the most used imaging modalities for guiding percutaneous interventions, but each has limitations that can affect the accuracy of the biopsy.

Percutaneous spine biopsy performed under fluoroscopy guidance has been extensively studied regarding its accuracy and adequacy. The reported accuracy of this procedure ranges between 16% and 92%, while the complication rates range from 0% to 10% [15]. Notably, Kamei et al. conducted a series of 128 cases and reported a high accuracy rate of 93.8% for percutaneous spine biopsy performed under fluoroscopy guidance [16]. Similarly, Dave et al. reported an adequacy rate of 88.7% for percutaneous spine biopsy conducted under fluoroscopy guidance [17]. These findings highlight the promising outcomes and reliability of fluoroscopy-guided percutaneous spine biopsy as a valuable diagnostic tool for obtaining accurate and adequate tissue samples.

Renfrew et al. [18] proposed that CT-guided percutaneous transpedicular spine biopsy is recommended in cases where the location of a vertebral body lesion makes it challenging to access using the posterolateral approach. This recommendation was due to the proximity of neural elements to the pedicle. However, with the availability of high-resolution image intensifiers, it is possible to visualize the vertebral aspects in sufficient detail, allowing for the protection of the medial and inferior walls of the pedicle during biopsy, thereby avoiding injury to neural elements. On the contrary, Dave et al. [17] did not observe any distinct advantages of CT guidance over image intensification. Furthermore, image intensification offers the benefits of maintaining an aseptic environment in the operating room and being more cost-effective than CT scans.

CT guidance has become the modality of choice due to its higher accuracy and reduced complications. However, the reliability of this technique varies between 70 and 93%. It is believed that these rates depend significantly on the level (cervical, dorsal, or lumbar) the biopsy was obtained from, the type (primary or secondary) of tumor targeting, and its nature (heterogenous or homogenous) [14,19,20,21,22]. Hence, a second needle biopsy, or incisional biopsy for the misdiagnosed lesions, is usually performed. 

Since MRI has a superior soft-tissue and bone marrow contrast [8,23] and some lesions such as metastatic multiple myeloma and non-Hodgkin’s lymphoma can only be visualized better by MRI [2], MRI guidance is becoming an accurate complementary diagnostic method to other imaging modalities for biopsy guidance spinal tumor biopsies [8]. Liu M et al. even argued that MR-guided spinal biopsy alone is enough and has accuracy rates higher than the CT-guided method [2].

Carrino J.A. et al. investigated the application of MRI in percutaneous biopsies of musculoskeletal lesions. The researchers detected a remarkable success rate in obtaining sufficient biopsy samples under MRI guidance. This approach proved to be effective in cases including small lesions or those situated in anatomically challenging regions, leading to enhanced diagnostic accuracy. Additionally, the study stressed the benefits associated with MRI-guided percutaneous biopsies. These findings contribute to the growing body of evidence supporting the value and reliability of MRI-guided percutaneous biopsy as a diagnostic tool for musculoskeletal lesions [8].

Our study shows a significant difference between MRI and CT modalities, particularly in the axial and sagittal planes. This highlights the superiority of MRI images in providing more reliable and detailed information, which is crucial for precise diagnosis and effective treatment planning. Moreover, these observations stress the essential role of MRI imaging for obtaining biopsy samples with the necessary accuracy and adequacy.

Integrating CBCT and MRI can be valuable in the post-chemotherapy or immunotherapy follow-up management of complex tumors. It is worth emphasizing that the histopathological assessment of giant cell tumor of bone (GCTB) following denosumab treatment should be approached cautiously due to the possibility of alterations in cell count and the emergence of new bone [24,25,26]. Nevertheless, certain studies have reported no substantial pathological changes upon histopathological analysis following denosumab treatment [27]. 

To increase diagnostic accuracy and reduce the need for additional invasive procedures, we examined the effectiveness of combining MRI and CT imaging modalities for directing spinal biopsies in 18 patients with different spinal pathologies. The findings of our study revealed that the combined technique accurately identified the diseased tissue type in all 18 biopsies. These results provide a solid foundation for considering alternative treatment options.

Our study leveraged the strengths of both MRI and CT imaging by utilizing MRI preoperatively and merging it with the intraoperative CT navigation system. This approach allowed for continuous monitoring of the biopsy process, ensuring accurate sampling of viable, non-necrotic tumor tissue. Additionally, comparing pre- and postoperative scans validated the biopsy outcomes. While our technique offers significant advantages, we acknowledge its limitations, such as the need for general anesthesia, time consumption, and the requirement of an expensive O-arm device that may not be accessible in all healthcare centers.

Considering these factors, we propose reserving this technique for smaller, more complex tumors or cases with anatomically challenging locations where the likelihood of complications is higher. By focusing on challenging scenarios, we can maximize the benefits of our approach and optimize its usage.

## 5. Conclusions

Our study supports the integration of CT and MRI imaging in spinal biopsies, leading to improved diagnostic accuracy and efficiency. However, larger-scale studies are needed to validate these findings and explore their benefits in different clinical scenarios. Although time-consuming and requiring general anesthesia, this approach helps avoid misdiagnosed lesions in complex tumors. Our findings emphasize the importance of selecting the appropriate imaging modality for accurate tumor size measurements. 

## Figures and Tables

**Table 1:** Summary of biopsy details and results.

Patient Number	Biopsy Level	Previous Cytology	Previously Diagnosed Tumor	O-Arm-MRI Navigated Biopsy Result
1	L III	Yes	FL	FL
2	S I	No	NK	FL
3	L III	Yes	NK	MGUS
4	D VIII	No	BC	BC
5	D VII	Yes	NK	BH
6	L V	No	NK	BSC
7	L V	Yes	FL	FL
8	D IX	No	DLBCL	DLBCL
9	D XI	No	BC	BC
10	D XII	Yes	DLBCL	DLBCL
11	L I	No	NK	MGUS
12	L V	No	BC and RCC	BC
13	L II	Yes	BC	BC
14	D VI	Yes	BC	BC
15	L V	No	PCa	PCa
16	D XII	No	BC and LUAD	LUAD
17	L V	No	BC and LUAD	BC
18	LV	No	OSCC	OSCC

Abbreviations: breast cancer (BC), lung adenocarcinoma (LUAD), ovarian squamous cell carcinoma (OSCC), prostate carcinoma (PCa), diffuse large B-cell lymphoma (DLBCL), benign hemangioma (BH), benign syovial cyst (BSC), follicular lymphoma (FL), monoclonal gammopathy of undetermined significance (MGUS), and not known (NK).

## Data Availability

All data generated or analyzed during this study are included in this published article.
